# Multi-model seascape genomics identifies distinct environmental drivers of selection among sympatric marine species

**DOI:** 10.1186/s12862-020-01679-4

**Published:** 2020-09-16

**Authors:** Erica S. Nielsen, Romina Henriques, Maria Beger, Robert J. Toonen, Sophie von der Heyden

**Affiliations:** 1 Evolutionary Genomics Group, Department of Botany and Zoology, University of Stellenbosch, Private Bag X1, Matieland, 7602 South Africa; 2 Technical University of Denmark, National Institute of Aquatic Resources, Section for Marine Living Resources, Velsøvej 39, 8600 Silkeborg, Denmark; 3 School of Biology, Faculty of Biological Sciences, University of Leeds, Leeds, LS2 9JT UK; 4 Hawaiʻi Institute of Marine Biology, University of Hawaiʻi at Mānoa, Kāne’ohe, HI 96744 USA

**Keywords:** Pool-seq, RAD-seq, Seascape genomics, Environmental association, Comparative phylogeography, Marine invertebrates

## Abstract

**Background:**

As global change and anthropogenic pressures continue to increase, conservation and management increasingly needs to consider species’ potential to adapt to novel environmental conditions. Therefore, it is imperative to characterise the main selective forces acting on ecosystems, and how these may influence the evolutionary potential of populations and species. Using a multi-model seascape genomics approach, we compare putative environmental drivers of selection in three sympatric southern African marine invertebrates with contrasting ecology and life histories: Cape urchin (*Parechinus angulosus*), Common shore crab (*Cyclograpsus punctatu*s), and Granular limpet (*Scutellastra granularis*).

**Results:**

Using pooled (Pool-seq), restriction-site associated DNA sequencing (RAD-seq), and seven outlier detection methods, we characterise genomic variation between populations along a strong biogeographical gradient. Of the three species, only *S. granularis* showed significant isolation-by-distance, and isolation-by-environment driven by sea surface temperatures (SST). In contrast, sea surface salinity (SSS) and range in air temperature correlated more strongly with genomic variation in *C. punctatus* and *P. angulosus*. Differences were also found in genomic structuring between the three species, with outlier loci contributing to two clusters in the East and West Coasts for *S. granularis* and *P. angulosus*, but not for *C. punctatus*.

**Conclusion:**

The findings illustrate distinct evolutionary potential across species, suggesting that species-specific habitat requirements and responses to environmental stresses may be better predictors of evolutionary patterns than the strong environmental gradients within the region. We also found large discrepancies between outlier detection methodologies, and thus offer a novel multi-model approach to identifying the principal environmental selection forces acting on species. Overall, this work highlights how adding a comparative approach to seascape genomics (both with multiple models and species) can elucidate the intricate evolutionary responses of ecosystems to global change.

## Background

Anthropogenic climate change is altering the physical and chemical properties of coastal ecosystems at an unprecedented rate, ultimately threatening the persistence of biological communities [1, 2]. Nearshore environments are especially at risk from anthropogenic change as they are exposed to threats from both the terrestrial and marine realms [3]. Coastal systems experience strong environmental gradients, caused by complex interactions among features such as wind and wave action, ocean currents and upwelling cells, and exposure to sunlight and precipitation [4]. Environmental heterogeneity in coastal systems should therefore impose differential selection pressures, facilitating local adaptation and genetic differentiation [5, 6]. While many marine species are thought to exhibit low genetic differentiation due to large population sizes and high dispersal potential, there is growing evidence suggesting that many coastal organisms display surprisingly fine-scale population structuring and local adaptation [7–10]. Along with oceanographic patterns and coastal topography, the support for climatic environmental gradients acting as barriers to gene flow is steadily increasing [11–14]. Uncovering patterns of genetic differentiation and possible local adaptation, and distinguishing which environmental conditions shape such patterns, is critical for effective conservation management in the face of global change [15–18].

Quantifying genomic differentiation and putative adaptive variation of marine species, and the resultant field of seascape genomics, relies on recent advances in Next Generation Sequencing (NGS [19, 20]). One of the main goals of seascape genomics is to use NGS to identify loci that differ significantly over environmental gradients, using gene-environment association analyses (GEAAs [21, 22]). GEAAs are powerful tools to detect putative adaptive loci (commonly termed ‘outlier loci’) by directly associating allele frequencies with environmental variables [5, 23, 24]. Sea surface temperature (SST) is the most common environmental structuring force identified among seascape studies to date [25], and has been shown to strongly correlate with genomic variation in abalone [14], mussels [26], oysters [12, 27], sea cucumbers [28], and lobsters [11]. As SST is consistently identified as one of the prominent drivers of genomic variation in marine invertebrates, it has promise as a proxy for evolutionary processes, such as local selection, in conservation [29]. However, previous studies have solely investigated single-species GEAAs, which means that the effects of SST and other environmental variables on coastal species with similar distributions, but different micro-environmental niches, are still largely unexplored [1]. Furthermore, there are a multitude of GEA methods available, which differ in their statistical analyses and assumptions of demographic histories, often leading to diverse outputs [30, 31]. Even though many studies use two or three outlier detection methods to account for false positives [23], there has yet to be a comprehensive comparison of various methods in their ability to identify the dominant selection forces acting on wild marine populations.

**Fig. 1:**
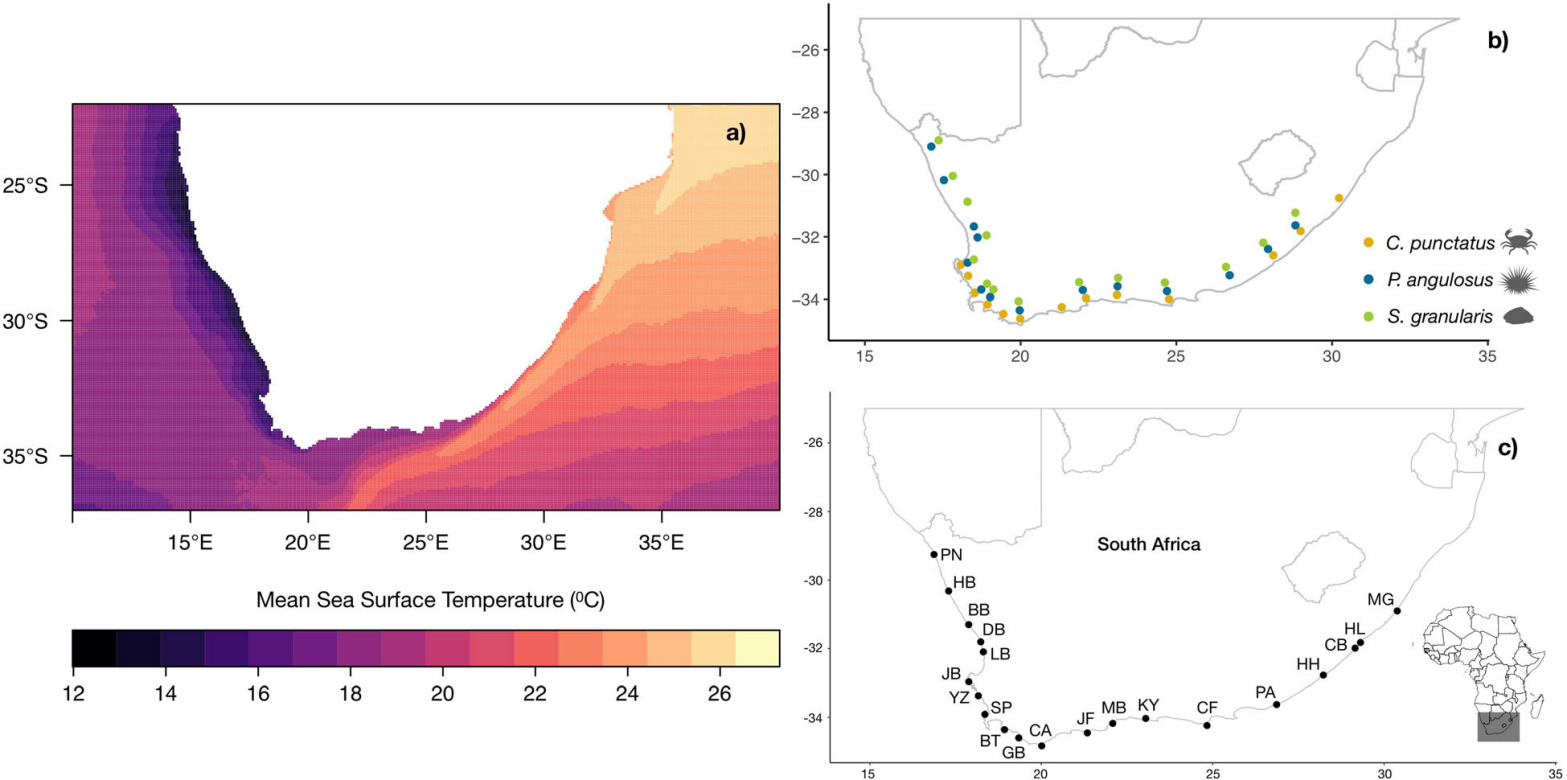
The Sea Surface Temperatures (from the MARSPEC database) within the study region (**a**), the species-specific sampling regimes (**b**), and the names of the 20 total sample sites included in the study (**c**)

This study focusses on the environmental drivers of genomic differentiation in three rocky shore invertebrates: Cape urchin (*Parechinus angulosus*), Granular limpet (*Scutellastra granularis*), and Common shore crab (*Cyclograpsus punctatus*), that are widely distributed along the southern African coastline, which is known for its strong biogeographic gradients of temperature, productivity and other environmental variables (Fig. 1 [32]). Previous studies, consisting of mitochondrial DNA (mtDNA) data, have detected multiple lineages for each species, broadly differentiated into West and East Coast clades [33–35], with evidence of isolation-by-distance (IBD; e.g. [35]). However, a recent study using NGS data from the estuarine-restricted seagrass *Z. capensis* suggested that isolation-by-environment (IBE) plays a significant role in shaping the genomic differentiation [36], although the extent that IBD and IBE characterise the genomic variation of other marine species in the region currently remains unknown.

Broadly, the objectives of this study are to characterise phylogeographic patterns of three ecologically important rocky shore species, and to identify the dominant environmental drivers of putative adaptive variation within southern African rocky shore communities. A pooled (Pool-seq), restriction-site associated DNA sequencing (RAD-seq) approach was used to characterise genomic variation across at least 13 sites per species, and describe population diversity within and differentiation amongst these species. Seven outlier detection methods were used to distinguish the principal environmental drivers of selection in each species. We hypothesized that: 1) each species will be structured into West and East Coast populations in accordance with mtDNA population structure, 2) each species will show significant isolation-by-distance and isolation-by-environment, 3) SST will be the dominant driver of putative selection for all three species.

## Results

### Sequencing and bioinformatic processing

DNA samples were collected from three species, including 13 or 14 sites each, per species (Fig. 1; Tables S1-S3, Additional file 1). A pooled ezRAD sequencing and de novo assembly approach was used, as this allowed for larger contig lengths (e.g. > 1000 base pairs; bp) compared to other RAD-seq approaches [37]. Further, the ezRAD approach does not rely on a PCR step to amplify sequences during library preparation, which removes potential biases from PCR duplicates, and is a unique RAD-seq method which allows for high coverage at specific RAD loci in combination with low coverage of across the entire genome [38]. Additionally, the effectiveness of ezRAD has been verified with Pool-seq, which is a cost-effective method of sequencing multiple individuals, and is increasingly being used to characterize population level differentiation [39, 40]. To avoid any potential sequencing biases, we followed best practices by including a large number of individuals per pool (~ 40), as well as using a stringent coverage cut-off of > 20X per pool [41]. Filtering was also conducted to obtain high-quality allele frequencies, such as only retaining reads with mapping quality > 20, and only keeping properly mapped, and properly paired mapped reads (Table S4, Additional file 1). Additional bioinformatic steps (see Methods section for further details) were used to obtain single nucleotide polymorphisms (SNPs), from which various comparative phylogeography analyses were performed.

To assure retained SNPs best reflect nuclear genome-wide variation, we first removed possible mtDNA reads, as well as compared the performance of three de novo assemblers. The average number of reads per pool that mapped onto the reference mitogenomes was 12,363 for *C. punctatus*, 20,342 for *P. angulosus*, and 234 for *S. granularis* (Table S5, Additional file 1). These mitochondrial reads were subsequently removed from the raw reads during the mapping stage, as they reflect distinct evolutionary processes compared to nuclear loci [42]. As there are no reference genomes for these or closely-related species, de novo assemblies were compared between three programs, SPAdes, AbySS, and MEGAHIT, for each species. There are multiple measures to asses de novo assemblies, and here we followed common practice of choosing the assembly with higher N50 and L50 values, and those with fewer but longer contig lengths [43]. De novo assemblies were also BLASTed to the NCBI database, but less weight was put on this analysis as it can be biased toward model genomes [44]. SPAdes resulted in the more robust assembly, with the longest contig length, N50, and L50, as well as a higher number of NCBI hits on average for all three species (Table S6, Additional file 1), and thus was used for all downstream analyses.

The number of raw reads per species ranged from ~ 29 million for *C. punctatus* to ~ 47 million for *P. angulosus* (Table S7, Additional file 1). The average number of raw reads per pool was ~ 2.2 million for *C. punctatus*, ~ 2.5 million for *S. granularis*, and ~ 3.5 million for *P. angulosus* (Tables S8–10, Additional file 1). A total of 17,309, 3946, and 10,416 SNPs were identified for each species, respectively (Table S7, Additional file 1). After filtering for biallelic SNPS and pruning the SNP datasets to one SNP per 1000 bp (to account for linkage disequilibrium; LD), *C. punctatus*, *P. angulosus*, and *S. granularis* had 1190, 822, and 1658 SNPs, respectively (Table S7, Additional file 1).

### Genomic structuring

To assess population structuring, all filtered and LD-pruned SNPs were used to calculate pairwise Weir and Cockerham’s *F*_*ST*_ values and Nei’s genetic distances. We further investigated population structure with scaled covariance (Ω) matrices produced by the BayPass v.2.1 core model, which explicitly accounts for Pool-seq data [45]. The scaled covariance matrix characterises the covariation of allele frequencies both within and between pools, and can be interpreted as pairwise relatedness estimates of population structure. Isolation-by-distance (IBD) patterns were assessed by comparing genomic and geographic distance per species.

**Fig. 2:**
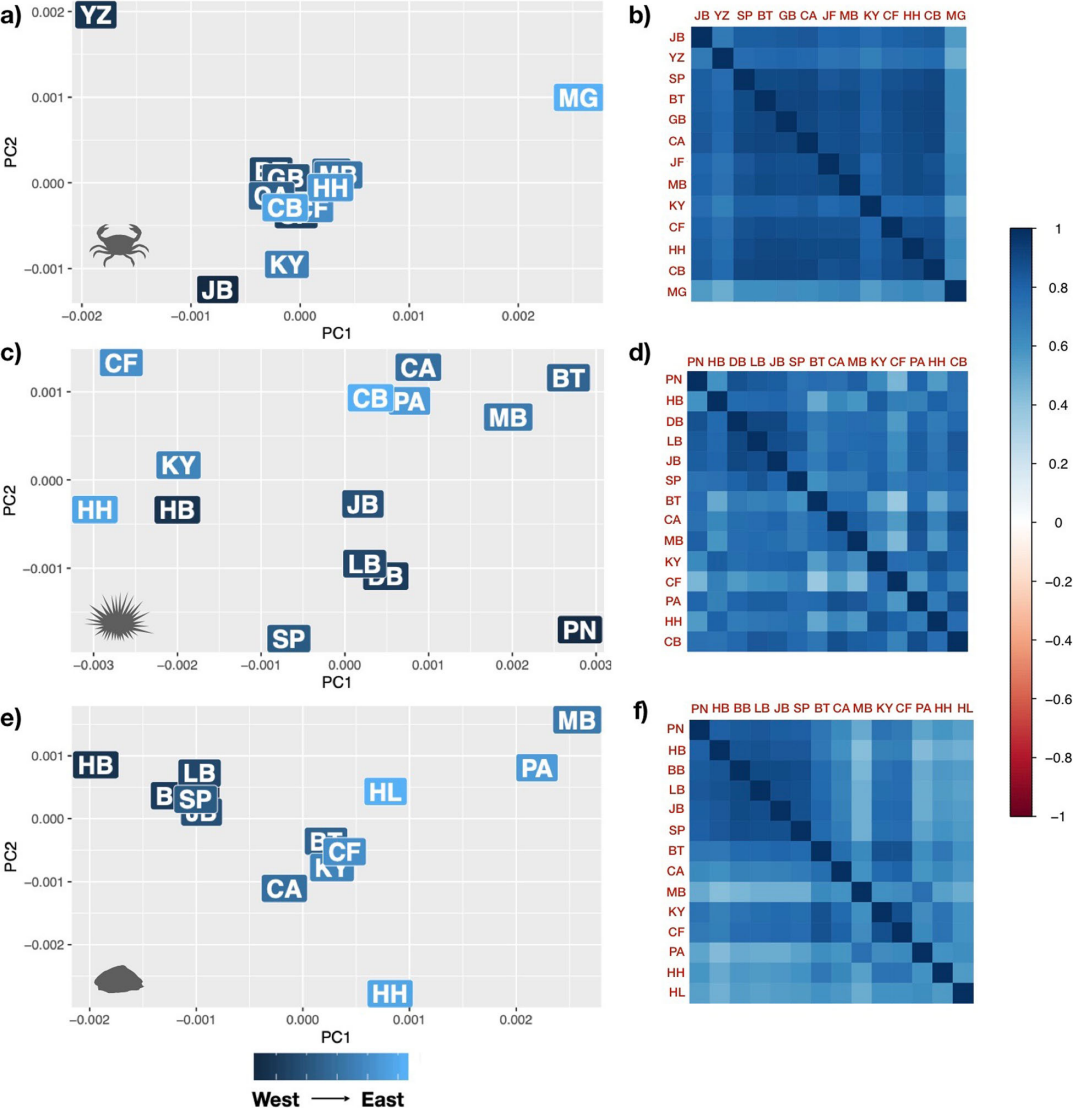
Population differentiation is shown by PCoAs of Nei’s genetic distance from all quality-filtered SNPs (**a**, **c**, **e**) and covariance (Ω) matrices represented as heatmaps (**b**, **d**, **f**), shown for *C. punctatus* (**a**, **b**), *P. angulosus* (**c**, **d**), and *S. granularis* (**e**, **f**). Letters in the PCoAs (**a**, **c**, **e**) correspond to the sample sites shown in Fig. 1, with darker shaded letters corresponding to western sites, and lighter shaded letters corresponding to eastern sites

Pairwise *F*_*ST*_ values varied between species, with ranges of: *C. punctatus F*_*ST*_ = 0–0.021, *P. angulosus F*_*ST*_ = 0–0.127, and *S. granularis F*_*ST*_ = 0 - *F*_*ST*_ = 0.059 (Additional file 2). The PCoAs from Nei’s genetic distance and the Ω heatmap matrices show no clear spatial clustering for *C. punctatus* and *P. angulosus*, but slight differentiation between West and East Coast sites for *S. granularis* (Fig. 2). Mantel tests suggest that of the three species, only *S. granularis* populations are characterised by IBD (*r* = 0.48, *p* < 0.01; Table 1).

**Table 1:** Mantel and partial Mantel test results for *C. punctatus*, *P. angulosus*, and *S. granularis*

	*C. punctatus*			*P. angulosus*			*S. granularis*		
Test	r	p	q	r	p	q	r	p	q
FST ~ D	0.18	0.20	0.39	0.13	0.18	0.54	0.48	**0.00**	**0.01**
FST ~ SSSmean|D	0.18	0.45	0.67	−0.02	0.87	0.87	−0.23	0.27	0.34
FST ~ SSSrange|D	−0.50	0.06	0.19	−0.02	0.77	0.87	−0.23	**0.03**	0.06
FST ~ SSTmean|D	0.43	**0.03**	0.19	−0.20	0.14	0.54	0.40	**0.00**	**0.01**
FST ~ SSTrange|D	0.04	0.79	0.79	−0.08	0.46	0.87	−0.14	0.39	0.39
FST ~ Trange|D	0.08	0.61	0.73	−0.06	0.71	0.87	−0.19	0.28	0.34

Correlation coefficients (r), *p-values* (p), and *q-values* (q) are given for tests between genetic distance (FST) and geographic distance (D), and distance matrices between each of the five environmental variables: mean sea surface salinity (SSSmean), sea surface salinity range (SSSrange), mean sea surface temperature (SSTmean), sea surface temperature range (SSTrange), and surface air temperature range (Trange). Significant values are denoted in bold

The results also show that altering coverage cut-off parameters has little influence on patterns of population structure, as the two-dimensional visualizations of genomic differentiation (derived from Ω matrices), show similar genomic clustering across three coverage scenarios per species (Fig. S2-S4, Additional file 1), confirming other studies which found that Pool-seq population differentiation patterns are robust to coverage variances [46, 47].

### Potential environmental drivers of genomic structuring

To assess possible environmental drivers of genomic structuring, we ran isolation-by-environment (IBE) tests, which compare genomic and environmental distance, accounting for geographic distance. To identify environmental variables for the IBE and GEA analyses, a total of 20 environmental variables were originally included, and subsequently filtered based on Spearman’s correlation coefficients < 0.65 and variance inflation factors < 10. There were multiple correlations between the 20 environmental predictor variables (Additional file 3). After filtering for collinearity, five final environmental predictor variables remained: mean sea surface salinity (SSSmean), sea surface salinity range (SSSrange), mean sea surface temperature (SSTmean), sea surface temperature range (SSTrange) and air temperature range (Trange; Additional file 3).

Partial Mantel tests showed significant IBE by SSTmean for *C. punctatus* (*r* = 0.43, *p* < 0.05), but this did not remain significant after correcting for multiple testing (q = 0.19; Table 1). SSTmean was also found to significantly correlate with genomic differentiation in *S. granularis* (*r* = 0.40, *p* < 0.01), which remained significant after multiple testing correction (q = 0.001; Table 1). The partial Mantel tests did not find a significant correlation between any of the three environmental predictor variables and genomic differentiation in *P. angulosus* (Table 1).

### Characterising possible selection signals via outlier loci identification

As GEAA methods have been shown to vary in the type and number of outliers detected [23, 30], seven different outlier-detection methods were compared, including six GEAAs to investigate possible associations between SNPs and environmental variables. The analyses included BayPass Bayesian hierarchical models (both core and auxiliary models), Latent factor mixed models (LFMM), Moran spectral outlier detection (MSOD) and Moran spectral randomization outlier detection (MSR), and Redundancy analyses (RDA) and Distance-based redundancy analyses (dbRDA).

Overall, there was a large range in the number of outliers detected, with little overlap between models (Table 2). LFMM detected the most outliers and had the highest number of unique outliers, followed by MSOD (Table 2). Generally, *S. granularis* had the highest number of outliers detected for each model, with the exception of LFMM (Table 2). The model type with the lowest number of outliers selected was dbRDA (Table 2). For the dbRDA analyses, a forward selection process retained zero dbMEMs for *C. punctatus* and *P. angulosus,* and one dbMEM for *S. granularis*. The dbRDA for *S. granularis* had an adjusted R^2^ value of 0.02 (*p* = 0.33), with one outlier locus selected. The standard RDAs had adjusted R^2^ values of 0.021 (*p* = 0.31), 0.021 (*p* = 0.65), and 0.084 (*p* = 0.01) for *C. punctatus*, *P. angulosus*, and *S. granularis*, respectively. The single population-differentiation based outlier detection method, BayPass core model (BPC), identified nine outliers for *C. punctatus*, five outliers in *P. angulosus*, and 19 in *S. granularis*, with two, two, and eight outliers unique to that method, respectively (Table 2).

**Table 2:** Comparisons in number of outlier SNPs detected between seven outlier detection methods

Method (Abbreviation)	Model type	Correction for spatial or population structure	*C. punctatus* # outliers (# unique)	*P. angulosus* # outliers (# unique)	*S. granularis* # outliers (# unique)
BayPass core model (BPC)	Bayesian	Yes, population	9 (2)	5 (0)	19 (5)
BayPass auxiliary model (BPA)	Bayesian	Yes, population	0	0	4 (0)
Latent factor mixed model (LFMM)	Mixed model	Yes, population	134 (121)	72 (60)	125 (101)
Moran spectral outlier detection (MSOD)	Multivariate model	Yes, spatial	15 (14)	9 (7)	20 (18)
Moran spectral randomization outlier detection (MSR)	Multivariate model	Yes, spatial	3 (NA)	3 (NA)	8 (NA)
Redundancy analysis (RDA)	Multivariate model	No	9 (3)	9 (1)	16 (2)
Distance-based redundancy analysis (dbRDA)	Multivariate model	Yes, spatial	0	0	1 (1)

Descriptions of outlier detection methods, and the number of total and unique outliers (restricted to that method) detected by each method for each species. Note that MSR could not have unique outliers as it uses those identified by MSOD

**Fig. 3:**
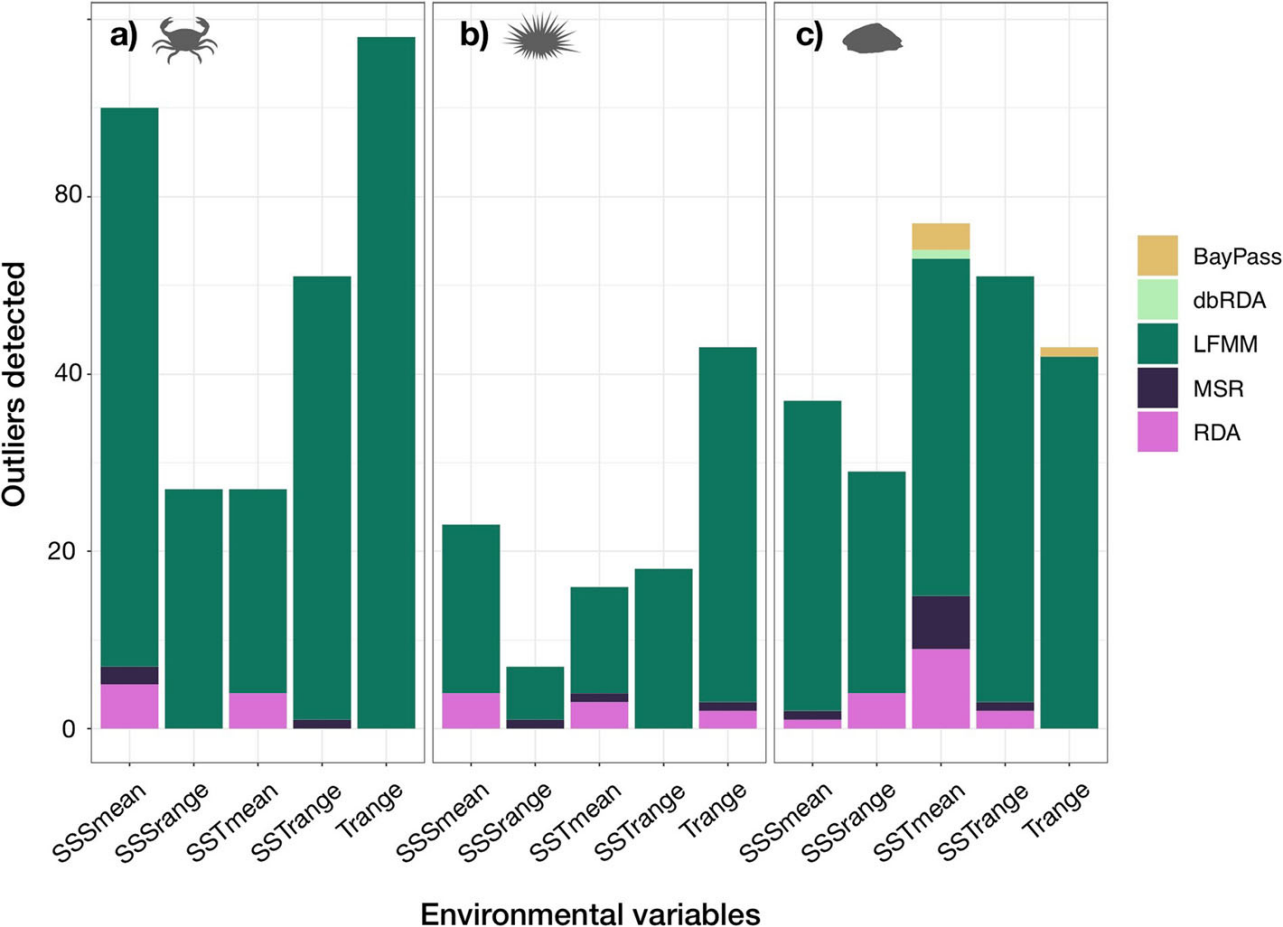
The number of outlier SNPs detected per method for *C. punctatus* (**a**), *P. angulosus* (**b**), and *S. granularis* (**c**). See Table 2 for method abbreviations

The environmental variable that most strongly correlates with genomic variation differed between outlier detection methods and across species. The majority of methods for *C. punctatus* identified the most outlier loci in association with SSSmean, with the exception of LFMM that identified the most outliers with Trange (Fig. 3). Trange and SSTmean were the two variables that identified outliers in at least three models for *P. angulosus* (Fig. 3). SSTmean identified the most outlier loci in all methods except LFMM for *S. granularis* (Fig. 3).

### Genomic structure of putatively neutral vs. outlier SNPs

Loci that were selected by two or more outlier detection methods (2X outliers) were used to create an ‘outlier dataset’, and these loci were removed from the total SNP dataset to create a ‘putatively neutral dataset’. We compared the genomic structuring between putative neutral and outlier SNPs via Principal Components Analysis (PCA) ordinations of allele frequencies from each dataset.

**Fig. 4:**
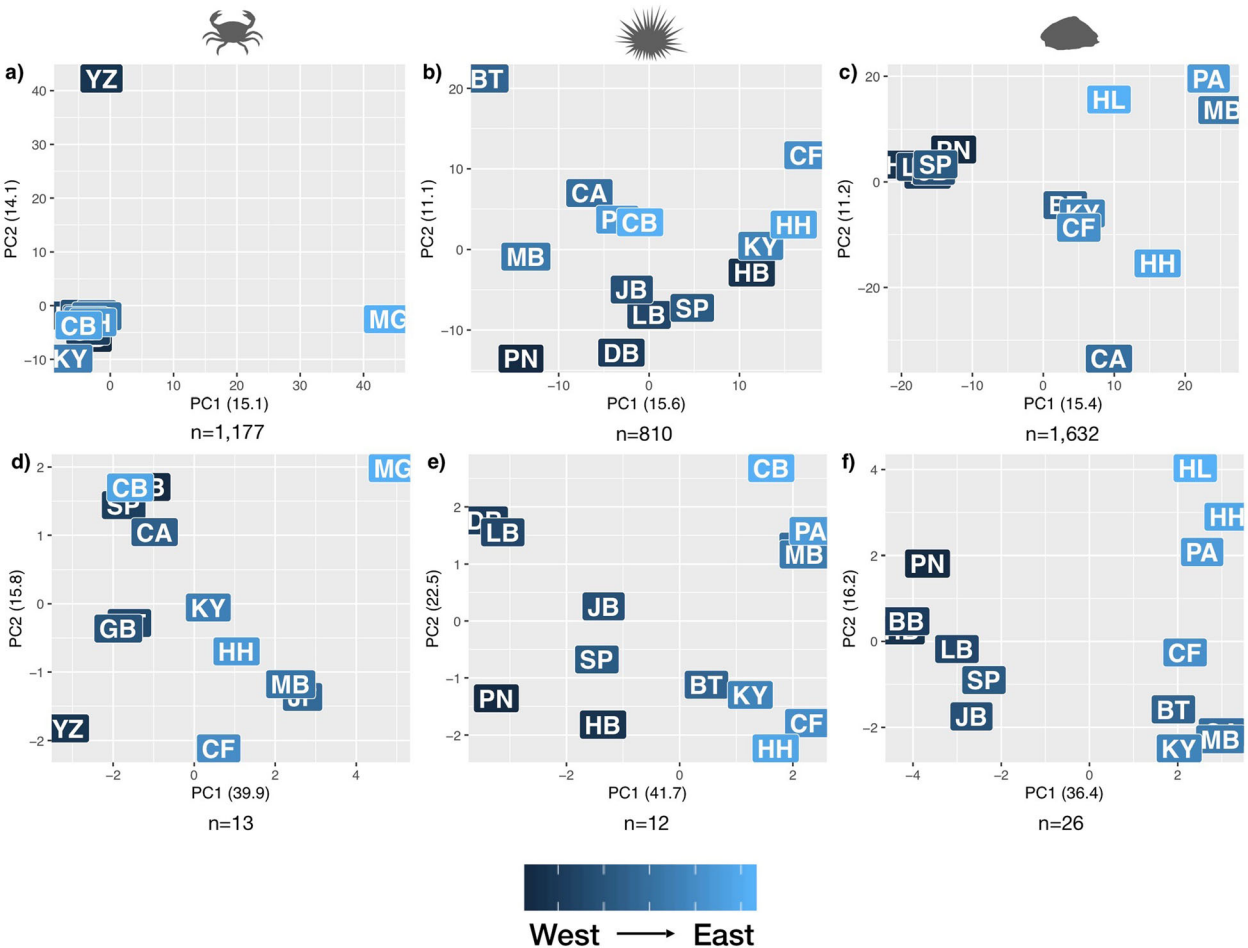
Genomic differentiation as shown by PCAs of allele frequencies in either the putatively neutral (**a**-**c**) or outlier (**d**-**f**) datasets for *C. punctatus* (**a**, **d**), *P. angulosus* (**b**, **e**), and *S. granularis* (**c**, **f**). Letters correspond to the sample sites shown in Fig. 1, with darker shaded letters corresponding to western sites, and lighter shaded letters corresponding to eastern sites

The number of SNPs used to create ‘outlier’ datasets was 13, 12, and 26 for *C. punctatus*, *P. angulosus*, and *S. granularis*, respectively. The PCAs of allele frequencies differed between the putatively neutral and outlier SNP datasets for all three species (Fig. 4). For *C. punctatus*, the putatively neutral SNPs show most of the sites within one main cluster, with the YZ and MG sample sites each forming individual clusters. In contrast, the outliers show more differentiation between sites, with MG and YZ as most divergent. The putatively neutral SNPs of *P. angulosus* do not separate sites following any geographical pattern, however the outlier SNPs clearly distinguish between the East and West Coast sites (Fig. 4). In *S. granularis,* the putatively neutral dataset separates East Coasts and West Coast sites, a pattern even more pronounced when examining the outlier dataset, where sampling sites are clearly differentiated according to geography (Fig. 4).

### Potential functionality of outlier SNPs

We investigated the potential functional roles of the outlier SNPs selected by two or more detection methods (2X outliers), by BLASTing them to the NCBI non-redundant protein sequence database, and assessing gene ontology (GO) with Blast2GO.

Of the 2X outliers, seven contigs from *C. punctatus*, six contigs from *P. angulosus,* and 11 contigs from *S. granularis* resulted in BLAST results passing quality filters (Additional file 4). Several contigs from *P. angulosus*, and *S. granularis* matched to histone complexes, with GO terms relating to DNA-binding, protein heterodimerization activity, and regulation of DNA recombination and chromatin silencing (Additional File 4). The remaining contigs with BLAST hits for *S. granularis* had GO terms relating to regulation of transcription, GTPase activity, and cell adhesion (Additional file 4). The GO terms relating to the BLAST hits for *C. punctatus* include protein and ion transport, carbohydrate metabolism, DNA binding and synthesis, and the anaphase-promoting complex (Additional file 4).

## Discussion

This study builds on single-marker genetic analyses, by utilising high-throughput genomic data to elucidate phylogeographic patterns of three southern African coastal marine invertebrates. We hypothesized that the study species would each demonstrate West and East Coast genomic structuring as well as isolation-by-distance (IBD) and isolation-by-environment (IBE). However, these hypotheses were only confirmed in the limpet, *S. granularis*. We also used a multispecies approach to explore putative environmental drivers of genomic variation within this unique marine biogeographical gradient. Here we hypothesized that sea-surface temperature (SST) would be the dominant driver of genomic variation, and yet again this hypothesis was rejected in all species except for *S. granularis*. These findings display how high-throughput sequencing can elucidate distinctive population structuring and gene-environment associations, and offer exciting avenues for future research which investigate these evolutionary processes at even finer scales. Overall, the results reveal species-specific evolutionary patterns, highlighting the complexity of interacting factors shaping natural genomic variation, which is discussed in detail in the following sections.

### Genomic markers elucidate distinct patterns of population structuring

Our first hypothesis was that each species would reflect previously described mtDNA patterns, with two clusters separated into West and East Coast individuals, reflecting the biogeographic breakpoint around the Southwestern Cape. However, only the limpet *S. granularis* follows this pattern, with *C. punctatus* showing high connectivity between populations, and *P. angulosus* showing no clear population structuring (Fig. 2; Additional file 2). The discordance in genomic differentiation found between mtDNA datasets in previous studies and the SNPs datasets here could be owing to the differences between the two marker types, as mtDNA markers are comprised of a single maternally inherited locus, while SNP markers represent a broad range of loci across the nuclear genome [42]. Additionally, mtDNA markers are expected to reflect relatively historical evolutionary events compared to the more contemporary processes captured by microsatellite and SNP markers [48].

*Scutellastra granularis* was also the only species which supported our hypothesis of IBD and IBE influencing genomic structure. This pattern of IBD and IBE in *S. granularis* could also result from repeated founder effects and allele surfing, caused by colonization generating an allele frequency gradient which co-varies with the environmental gradient [49, 50]. However, *S. granularis* and *P. angulosus* were shown to have similar evolutionary histories [33, 34], and thus it seems more likely that contemporary environmental, rather than historical demographic, processes are leading to the distinct patterns found in *S. granularis*. The distinct IBD and IBE patterns found in *S. granularis* could be owing to this species having the shortest pelagic larval duration of the three study species, or due to it being a habitat specialist, preferring sheltered boulder shores [51]. Additionally, *S. granularis* was found to have the lowest thermal tolerance of four co-occurring mid-shore limpets [52], indicating that it may be highly sensitive to the temperature gradient within the region.

Even though *S. granularis* is the only species to show distinct West/East Coast structuring in all SNPs, both *P. angulosus* and *S. granularis* show strong West and East Coast clustering when using only outlier SNPs (Fig. 4). The West and East Coast bioregions exhibit profound differences in not only temperature, but other environmental variables such as primary productivity [53], which can potentially lead to local selection despite high levels of connectivity [54]. This finding builds on multiple other studies which have found outlier SNPs showing fine-scale genomic structuring in populations characterised by high genetic connectivity, yet situated within strong environmental gradients [14, 55, 56], and suggests that environmental variation along coastal South Africa plays an important role in the evolutionary dynamics of species in the region.

In contrast to the other two species, the crab *C. punctatus*, did not show a strong separation between the West and East Coast sites. Instead, two range-edge sampling locations (YZ and MG) are highly differentiated in both the putatively neutral and outlier SNP datasets (Fig. 4). It could be that this species is less affected by large-scale environmental gradients of the coastline, but rather that an edge effect driven by demographic processes explains this pattern [57]. Populations on the edge of a species’ distribution are generally more likely to experience historical distributional range shifts, which in combination with contemporary gene flow patterns, can explain the differentiation of these two populations in the putatively neutral SNPs [56, 57]. In addition, these sites are also on the edges of the species’ ecological niche, and may experience unique environmental variation, potentially leading to the increased differentiation seen in the outlier SNPs [58, 59]. There may also be selection forces specific to these two populations which were not included in the GEAAs, but which explain evolutionary dynamics of this species [25]. Ultimately, more comprehensive genomic data, such as whole-genome sequencing, and increased fine-scale ecological and environmental assessments are needed to confidently assess the unique population variation seen in *C. punctatus*.

The distinct distribution of genomic variation of *C. punctatus* could also result from it being the most generalist of the three species, inhabiting both estuarine and marine environments [60, 61]. A previous study by [62] found that among 10 rocky intertidal invertebrates, the ability to utilize sheltered habitat was the strongest predictor of genetic structure. Of the three species here, *S. granularis* is the most restricted in its habitat, compared to *P. angulosus* and *C. punctatus* which are able to shelter under rocks and macroaglae, rather than remain exposed on rocky surfaces [61]. While dispersal abilities could also potentially be driving intraspecific differences in population structure, it is unlikely that Pelagic Larval Duration (PLD) is driving this pattern, as *S. granularis* and *C. punctatus* have similar PLDs (~ 5–15 days) compared to *P. angulosus* (~ 50 days [35]), yet the patterns of genomic differentiation do not reflect the PLD similarities between species. However, additional ecological characteristics pertaining to the larval stages of each species, such as the effect of temperature on larval development [63, 64], in combination with the spatial and temporal variation in near-shore currents affecting larval dispersal [65], could be influencing population structure. Broadly, while the results show that each species exhibits weak levels of genomic variation, possibly owing to high levels of gene flow, there are fine-scale interspecific differences in genomic variation, which appear to vary based on the ecology of the species.

### Identifying drivers of selection using a multi-model approach

The number of outlier loci, as well as the environmental variable most strongly associated with outlier loci, varied across GEAA methodologies (Table 2; Fig. 3), which mirrors previous studies describing differences in outlier detection methods (e.g., [22, 30, 31]). Each GEAA accounts for demographic histories differently, leading to differences in outliers detected [31, 66], and thus it has been suggested to use multiple models in combination when the principal environmental variables are unknown [23]. At present, most studies use one or two outlier detection methods, and identify the most important environmental drivers of selection based on which variable identifies the most outlier SNPs [23]. However, our results, as well as those from [30], indicate that the number of SNPs identified as outliers varies greatly among detection methods. We argue that the importance of environmental variables should not be measured by the total number of outliers it detects, but rather by the number of GEAA approaches in which the variable identifies outliers. For example, even though Trange identified the most outliers for *C. punctatus*, we argue that the most important environmental driver of genetic differentiation for *C. punctatus* is SSS, because it identified outliers by three detection methods rather than only one for Trange (Fig. 3). Given that outlier detection methods are highly variable and subject to false positives [31], we believe that using multi-model approaches will increase the robustness of GEAAs, especially in studies identifying potential drivers of selection across species with varying evolutionary histories. Hence, in the following section, we discuss the dominant environmental drivers identified for each species based on the number of GEAA models in which outliers were identified.

### Different environmental drivers of selection across species

Of the three species, only *S. granularis* supported our hypothesis of SST being the most important environmental predictor of genomic variation (Fig. 3). Previous seascape genomic studies in temperate regions have frequently identified some measure of SST as the best predictor of genomic variation of marine invertebrates [9, 11, 12, 25, 28, 67], which is most likely due to SST affecting both cellular processes, and life-history events such as spawning and larval development [63]. However, for *P. angulosus,* Trange and SSTmean best explained genomic variation, whereas SSSmean best explained the structure of *C. punctatus*. Salinity emerging as a major selective force on *C. punctatus* is understandable, as this species is an osmoconformer that inhabits estuarine environments [68], and because the larval development of decapods is influenced by changes in salinity [69]. The lack of clear correlations with any environmental variables is unexpected for the urchin *P. angulosus*, given that previous studies have shown genomic variation corresponding to SST gradients in other echinoderms [28, 70]. Additionally, the paucity of annotated genomes for marine invertebrates makes it difficult to identify the functionality of outlier loci, which likely led to the limited number of BLAST hits for the 2X outliers in each species (Additional file 4). Despite this limitation, outliers from all species indicated a relation to DNA processing, which has previously been highlighted as a response to environmental pressures in marine invertebrates [71]. However, other than DNA synthesis and binding, the outliers BLASTed to a variety of proteins, involved in biological processes such as metabolism, cell adhesion, and protein transport (Additional file 4). Overall, the Gene Ontology results suggest that the biological pathways influenced in gene-environment interactions are species-specific, yet further work including more complete genomic information is needed to uncover the environmental footprints on the biology of these species.

Previous terrestrial comparative GEAA studies have found distinct results in co-occurring species, which the authors attribute to either differences in ecological niche ranges [72] or phenotypic plasticity [50]. A multitude of factors could be driving the interspecific differences observed here, as the study species not only inhabit different ecological niches, but also exhibit differential behaviours to remain within their physiological niches [61]. It is also likely that the study species exhibit phenotypic plasticity in response to environmental pressures, as plasticity and epigenetic effects have been noted in response to temperature and salinity at multiple life stages in marine invertebrates [73]. Additionally, the rocky shore is a highly variable environment, and it is likely that species within different zonations are under differential selection pressures at fine spatial scales [74, 75], which might interact with large-scale environmental gradients to create complex patterns of genomic variation.

## Conclusions

The results of our comparative genomic study suggest that environmental drivers, and the impacts from their future change, may be highly species-specific, even among co-occurring species living within regions of strong environmental gradients. Further, the results contrast many single-species marine GEAA studies by showing that SST does not consistently emerge as an important environmental force structuring the distribution of genomic variation in marine organisms. This finding brings into question the use of SST clines as simple surrogates for selection in marine conservation spatial planning with regards to global change. Yet the results here provide exciting opportunities to investigate the relationships between ecological or behavioural traits and environmental drivers of selection across species, which can be further assessed with common garden or physiological experiments.

This is one of the first comparative seascape genomic studies to date, and it is imperative that future seascape genomic studies aim to understand how climatic change will impact not just individual species, but communities [76]. Multispecies GEAA studies remain a challenge due to costs associated with high-throughput sequencing and the lack of annotated genomes in non-model species, particularly marine invertebrates [77, 78]. Here we used a pooled RAD-seq approach, which allowed us to conduct a multispecies comparative GEAA study with relatively low costs, albeit with some limitations such as low coverage alleles being lost due to filtering sequencing errors, and the identification of individuals and polygenic scores being unavailable [79, 80]. Additionally, using a reduced representation sequencing approach such as RAD-seq means that portions of the genome remain unknown, and many adaptive loci may not be captured [79]. However, for our research question, a pooled RAD-seq approach is beneficial as it allowed us to maximize the number of individuals per location to obtain accurate population allele frequency estimates [41, 81], as well as maximize the number of sample sites, both of which are essential for GEAAs [66, 82], without the full cost of sequencing every individual. As this study is a first step in elucidating the putative adaptive potential of coastal invertebrate species in this unique marine realm, further studies using more complete genomic sequencing strategies are needed to characterize the full breadth of selection processes. Finally, we also provide a novel approach to identify drivers of selection across a diverse array of species, by using multiple GEAA methods and inferring the importance of each variable across methods. Ultimately, we argue that future seascape genomics studies can benefit from widening their scope with species and model comparisons, to more robustly identify environmental drivers of selection.

## Methods

### Study region and species

The study domain lies along the South African coastline, which is one of the most biodiverse marine systems in the world [83]. This region has also been identified as hotspot for ocean warming as it is experiencing environmental change at a faster rate than predicted [84]. In South Africa, the coastline is characterised by SST increasing with longitude, from the cool-temperate Benguela region on the West Coast to the sup-tropical Dalgoa region on the East Coast (Fig. 1).

The study species were selected as their distributions span several bioregions and the natural environmental gradients of southern Africa (Fig. S1, Additional file 1), and can represent the high- (*C. punctatus*), mid- (*S. granularis*), and low- (*P. angulosus*) rocky shore ecotypes [62]. They also differ in life history traits with *C. punctatus* being a brooder, and *S. granularis* and *P. angulosus* being broadcast spawners, with PLDs varying from ~ 5–15 days (*S. granularis* and *C. punctatus*) to potentially up to 50 days (*P. angulosus* [34, 35, 85]). These species are each ecologically important; either as dominant grazers or scavengers, as substrates for other species to either live on, or as shelter for juvenile abalone [61].

A total of 14 sites, spanning ~ 2200 km of the South African coastline, were sampled for *S. granularis* and *P. angulosus*, and 13 sites spanning ~ 1800 km were sampled for *C. punctatus* (Fig. 1). These sites incorporate the natural environmental (e.g. SST, salinity, air temperature) gradients in the region, as well as the distributional range per study species [60].

### Laboratory protocols and bioinformatics

Genomic data consisted of pooled ezRAD-seq samples, as it is a cost-effective approach to obtain precise allele frequency data [41]. Dorant et al. (2019 [81]) found that Pool-seq inflated *F*_*ST*_ values relative to individual-based sequencing approaches, but still gave highly similar allele frequency outputs and patterns of population structure. Thus, while the absolute magnitude of *F*_*ST*_ values may be upwardly biased relative to sequencing individuals, for a fraction of the cost Pool-seq data still allow us to infer relative patterns of population structure with confidence [86].

Genomic RAD-seq data was previously obtained for the study species from 11 of the 20 sample sites ([87]; L. Mertens pers. comm.). Additional sampling was conducted at the remaining sites during July 2018, with 30–40 individuals collected from each site (Tables S1-S3, Additional file 1). Individuals were preserved in 100% ethanol, from which < 25 mg tissue (gonad from *P. angulosus*, foot from *S. granularis*, and muscle from *C. punctatus*) was taken for DNA extractions. Extractions were performed with the Qiagen DNeasy Blood & Tissue kit following the manufacturer’s protocols. The quality of the DNA extractions was assessed on 1% agarose gels and quantity was determined using the Qubit Quant iT dsDNA HS Assay system at the Central Analytical Facility at Stellenbosch University (CAF-SU). All extractions passing quality and quantity checks were stored at -20 °C. For each species, equimolar amounts of DNA from each individual were pooled per sample site, flash frozen and sent to the Hawaii Institute of Marine Biology (HIMB) for library preparation following [88] (further outlined in [89]). Equimolar pooled ezRAD libraries [37] were sequenced (V3, 2x300PE) on the Illumina Mi-Seq platform at University of California, Riverside.

The quality of raw FASTA reads were viewed with FastQC [89], and then uploaded onto the CAF-SU high performance cluster (HPC) for further analyses (see Table S4, Additional file 1 for outline of analyses). Bases with low quality scores (Q < 20), overrepresented sequences and adapter sequences were removed using TrimGalore! [90].

As mitochondrial DNA (mtDNA) markers have different evolutionary characteristics than nuclear markers [48, 91], we chose to filter mtDNA-mapped reads from the datasets [56]. In order to separate mtDNA from nuclear sequences, the quality-trimmed reads were first mapped onto mitogenome references of closely related species (Purple mottled shore crab, *Cyclograpsus granulosus*, NC_025571.1; Rea sea urchin, *Loxechinus albus*, JX888466.1; Fingered limpet, *Lottia digitalis*, DQ238599.1) using BWA-MEM ([92]; Table S5, Additional file 1). The mapped reads were converted to BAM files, sorted and filtered using SAMtools v.1.3 [93], and then merged using BAMtools [94]. The merged BAM files were converted back to SAM and used to filter the quality-trimmed reads, removing putative mtDNA markers before mapping, using the ‘filterbyname’ command in BBMap [95].

Given that there are no reference genomes for these or closely-related species, de novo assemblies were created, using quality-trimmed reads that were normalized to a coverage of 100X with BBMap ‘bbnorm’, and using k-mer value ranges identified with K-mer Genie [96]. The reads were assembled with three different programs: ABySS [97], MEGAHIT [98] and SPAdes [99]. Because SPAdes can only handle nine input samples at a time, we assembled half of each species’ samples at a time, and then merged the two SPAdes assemblies using GARM [100]. The outputs of the three assemblers were compared using QUAST v.4.1.1 [101] and the NCBI BLASTN v.2.4.0+ algorithm [102]. Metrics such as N50 and L50 values, and number of BLAST hits, were used to select a de novo assembly for further analysis.

We also mapped mtDNA-filtered reads to available reference genomes of the Purple urchin (GCA_000002235.4; 990.915 Mb), Owl limpet (GCA_000327385.1; 359.506 Mb), and Chinese mitten crab (GCA_003336515.1; 258.8 Gb) for comparison. Because these species are distantly related to our focal taxa, we had to relax SNP calling parameters (mapping quality > 10, minimum pool coverage = 10), but found that overall patterns of population structure were consistent between both approaches, mirroring the findings of [46]. As de novo assemblies have been shown to lead to more robust inferences than mapping onto loosely related genomes [103], we present only the more stringent de novo assembly approach here.

The mtDNA-free, but not normalized, reads were mapped onto the de novo assemblies with BWA-MEM. The subsequent SAM files were converted into BAM files, sorted, indexed and filtered with SAMtools. To control for sequencing biases, we down-sampled SAM files to the median number of reads across all pools with SAMtools. A synchronized multiple pileup file was created for each species with SAMtools ‘mpileup’, followed by the Popoolation2 ‘mpileup2sync.jar’ commands [104]. Final SNP calling was performed with the ‘popsync2pooldata’ function of the *poolfstat* v.0.0.1 R package [47]. To avoid potential biases associated with unequal sequencing of individuals within the pool, and since fewer SNPs at higher coverage has been shown to be more effective than a greater number of SNPs at lower coverage [105], we chose stringent SNP calling parameters of: minimum coverage >20X, minimum read count > 4, maximum read count <400X, and a minor allele frequency (MAF) > 0.01 in each pool [47, 81, 106]. To account for the possibility of loci being physically linked (linkage disequilibrium: LD) we further used custom R scripts to randomly select one SNP per 1000 bp per contig.

#### Assessing gene flow and potential drivers of population structuring

##### Characterising genomic differentiation

To assess genomic population structuring, pairwise Weir and Cockerham’s *F*_*ST*_ values from the LD-pruned SNP dataset were calculated using the ‘computeFST’ function of *poolfstat*, the confidence interval (CI) values of which were computed with a custom bash script from [81] using 1000 bootstrap iterations. Nei’s genetic distances matrices were generated with the ‘stamppNeisD’ function of the R package *StAMPP*, and visualized in Principal Coordinates Analyses (PCoAs) generated with the ‘pco’ function in the *ecodist* R package [107].

Additionally, the allele frequencies of all SNPs per species were input into the core model of BayPass v.2.1 [45] to estimate scaled covariance (Ω) matrices. BayPass is specifically designed to handle Pool-seq data, and uses allele-frequencies to create an Ω matrix, which can be interpreted as pairwise estimates of differentiation and population structure. BayPass was run under default conditions to create the Ω matrices, which were then converted into a correlation matrices using the ‘cov2cor’ function in R *stats* package, and visualized as similarity matrix heatmaps.

We also ran additional population structuring analyses to test if altering coverage cut-off parameters influences genomic differentiation patterns. To do so, we used subsets the LD-filtered SNP dataset described above, which underwent additional coverage filters of either: 1) maximum coverage < 200, or 2) minimum coverage > 40. We subsequently assessed how the different coverage scenarios influenced population structure by performing a singular value decomposition of the Ω matrices (from the core BayPass model) per scenario per species [81, 106].

##### Seascape features

The various seascape genomic analyses included a standard set of environmental features as predictor variables. A total of 20 environmental features were considered (Additional file 3), including air temperature and precipitation of the coldest month, warmest month, the range between coldest and warmest months, as well as annual mean between 1970 and 2000, which were downloaded from the WorldClim database at a resolution of ~ 1 km [108]. Annual mean, coldest ice-free month, and warmest ice-free month, and the range in SST between 2002 and 2010 and annual mean, monthly minimum and maximum, and range in sea surface salinity between 1955 and 2006 were downloaded from the MARSPEC database at a resolution of ~ 1 km [109]. Mean surface dissolved oxygen, diffuse attenuation coefficient, pH, and chlorophyll concentration between 2000 and 2014 were downloaded from the BIO-ORACLE database at a resolution of ~ 9.2 km [110]. Environmental features were downloaded for each sample site with the ‘load_layers’ function of the *sdmpredictors* R package [111]. We tested collinearity between predictor variables using pairwise Spearman’s correlation coefficients and Benjamini-Hochberg (BH) corrected *p*-values (*p* < 0.05 [112]). We removed variables that were significantly correlated (r > 0.65), and those with a variance inflation factor (VIF) > 10.

##### Isolation-by-distance (IBD) versus isolation-by-environment (IBE)

Isolation-by-distance (IBD) and isolation-by-environment (IBE) were tested using Mantel tests. Mantel tests are widely used in landscape genetics to test which spatial features are significant drivers of genetic differentiation [113]. IBD was assessed with a standard Mantel test, which evaluates the relationship between two matrices (i.e. geographic versus genetic distances) and IBE was tested with Partial Mantel tests, which compare the relationship between two matrices while taking into account the effect of a third (i.e. temperature versus genetic distance, accounting for geographic distance [113]).

IBD analyses consisted of Slatkin’s linearized pairwise *F*_*ST*_ (*F*_*ST*_ = [*F*_*ST*_ /(1-*F*_*ST*_)] [114]), and log-transformed geographic distances along the coastline calculated with the roadmap tool in QGIS [115], starting from the western-most site for each species. IBE analyses additionally included pairwise Euclidean climatic distances. Partial Mantel tests were performed for each climatic variable separately, with geographic distance as a conditioning variable. Individual Mantel test significance was assessed in *ecodist*, using 1000 permutations. To account for multiple tests, *p-* were converted to *q-values* and significance was assessed using a False Discovery Rate of 0.05 (FDR) based on BH criteria with the *qvalue* R package [116].

#### A multi-model approach to identifying environmental associations with SNPs

To investigate possible associations between SNPs and environmental variables, we used seven different outlier detection methods, using the same seascape features as stated above as predictor variables. As GEAA methods have been shown to vary in the type and number of outliers detected [23, 30], the multi-model approach used here allows for more robust inferences. The protocol pertaining to each outlier detection method are outlined below.

##### BayPass Bayesian hierarchical models

For an *F*_*ST*_-like outlier detection approach, the core model of BayPass was run, which uses a hierarchical Bayesian model to create per-locus *XTX* values, which can be interpreted as an *F*_*ST*_ values corrected for the scaled covariance (Ω) of population allele frequencies [45]. BayPass v.2.1 was run under default conditions to create *XTX* values. As described in [45], a pseudo-observed dataset (POD) was created to estimate the posterior predictive distribution of *XTX* values, and candidate SNPs were selected if they fell within the 99.9% quantile of the POD *XTX* distribution.

For a GEAA-like approach, the auxiliary model in BayPass was run to identify candidate SNPs due to associations with environmental variables. The auxiliary covariate model includes a binary auxiliary variable to classify the association and compute a Bayes factor (BF) for each locus while accounting for multiple testing [45]. After running the model under default conditions, we followed the general rule derived from [117], which identifies outliers as those having a log10 Bayes factor (db) > 20 [45].

##### Latent factor mixed models (LFMM)

Latent factor mixed models (LFMM) use mixed linear models to test for correlation between allele frequencies and an environmental predictor variable while correcting for population structure with latent factors [118]. As such, these models require a priori knowledge of the number of genetic clusters (K). K was inferred from previous mtDNA clustering analyses (K = 2 for each species [33–35]), as it is recommended to estimate K from independent genetic datasets [118]. LFMMs were run separately for each environmental variable using the R package *LEA* [119] with 10,000 cycles of the Gibbs sampling algorithm, 5000 burn-in cycles, and 10 replicate runs. For all runs per predictor variable, z-scores were combined, genomic inflation factor was calculated, and candidate loci were selected following using R scripts available from: http://membres-timc.imag.fr/Olivier.Francois/LEA.

##### Moran spectral outlier detection (MSOD) & Moran spectral randomization (MSR)

Moran spectral outlier detection (MSOD) uses Moran’s eigenvector maps (MEMs) to create power spectrums for each individual SNP, by taking the squared correlation coefficient of allele frequencies with MEM eigenvectors [120]. Candidate SNPs are then identified as having power spectra outside of the average spectrum across all SNPs. Moran spectral randomization (MSR), is then used to identify candidate SNPs that show a strong correlation to environmental variables by building the observed spatial structure into the null model, while accounting for spatial autocorrelation [120].

MEM axes were first created from geographic coordinates using the *spdep* R package [121], then power spectra corresponding MAFs and MEMs at each site were calculated. Z-scores were calculated for each locus based on the deviation from the average power spectrum following R code from: https://popgen.nescent.org/2016-12-13_MEM_outlier.html. The outlier loci identified by MSOD were then subjected to the MSR randomization approach, which tests the correlation between outlier MAFs and environmental variables, given the power spectra of each SNP. Using the *adespatial* R package, the MSR was run individually for each environmental variable, with 1000 permutations. We followed the suggested cut-offs of [120] of 0.01 and 0.05 for MSOD and MSR candidates, respectively.

##### Redundancy analysis (RDA)

Redundancy analyses (RDAs) are an extension of linear regressions that compare a matrix of dependent variables with multiple independent predictor variables. Linear regressions are calculated between allele frequencies and the climate variables at each site, while the fitted values are simultaneously constrained using a PCA. Environmental variables were centred and scaled, and allele frequencies were Hellinger transformed [122]. All RDAs were performed with the ‘rda’ function of the *vegan* R package [123]. Significance was assessed from the adjusted R^2^ value and with an ANOVA following 1000 permutations. Candidate loci were those that had loading scores ±3 Standard Deviations (SD) of the mean loading for each of the first two constrained axes [28, 30].

Distance-based RDAs (dbRDAs) were also run to account for autocorrelation between environmental and geographic distance. Distance-based Moran’s eigenvector maps (dbMEMS), which decompose Euclidean distances into a set of spatial variables [124], were created with the R package *adespatial* [125]. Significant dbMEMs were selected by first running an RDA solely using the dbMEMs as predictor variables, then using the adjusted R^2^ value from that RDA as the threshold for the forward selection procedure with the ‘forward.sel’ function in the *packfor* R package [126].

##### Outlier variation and functional annotation

Loci that were selected by two or more detection methods (2X outliers) were used to create a statistical ‘outlier dataset’, and these loci were removed from the total SNP dataset to create a ‘putatively neutral dataset’. Intraspecific outlier and putatively neutral variation was compared by running PCA ordinations on the MAFs of each dataset with the *vegan* package, and plotting the ordinations with the *ggplot2* package in Rstudio [127].

Furthermore, we investigated the potential functional roles of outlier SNPs selected by two or more detection methods (2X outliers). The contigs containing the 2X outliers were BLASTed against NCBI non-redundant protein sequence database for crustaceans (for *C. punctatus*), molluscs (for *S. granularis*), and sea urchins (for *P. angulosus*) using Blast2GO [128]. Search results were filtered to only include those which had an E-value less than 10^− 4^, and a minimal alignment length of 20 bp. Gene Ontology (GO) mapping and annotation was conducted on BLAST searches passing quality filters, using default parameters in Blast2GO.

## Supplementary information

**Additional file 1.** Sampling information, bioinformatic pipeline parameters, results of mitogenome mapping and de novo assembly comparisons, and single nucleotide polymorphism (SNP) results are shown per species. The study species distributions, and population clustering based on three coverage cut-off scenarios are also shown.

**Additional file 2. **Per species pairwise Weir and Cockerham’s *F*_*ST*_ and Nei's genetic distance values.

**Additional file 3. **The seascape features considered in the gene-environment association analyses, shown as values per sample site, as well as the Spearman’s R coefficients and *p*-values between variables.

**Additional file 4.** BLAST results against NCBI non-redundant protein sequences, including the species, contig ID and length, protein match, E-value, and mean percent identical. The Gene Ontology (GO) terms from Blast2GO are also shown.

## Data Availability

Raw reads accessible via Genbank accessions PRJNA660200 and PRJNA411764. Allele frequencies and R scripts accessible via GitHub: https://github.com/vonderHeydenLab/Nielsen_et_al_2020_BMC_Evol_Biol

## Abbreviations

2X outliers: outliers selected by two or more outlier-detection methods
BF: Bayes factor
BH: Benjamini-Hochberg
BPA: BayPass auxiliary model
BPC: BayPass core model
CAF-SU: Central Analytical Facility at Stellenbosch University
CI: confidence interval
dbRDA: distance-based redundancy analysis
GEAA: gene-environment association analysis
GO: gene ontology
HPC: high performance cluster
IBD: isolation-by-distance
IBE: isolation-by-environment
LD: linkage disequilibrium
LFMM: latent factor mixed models
MAF: minor allele frequency
MEM: Moran’s eigenvector map
MSOD: Moran spectral outlier detection
mtDNA: mitochondrial DNA
NGS: next generation sequencing
PCA: principal components analysis
PCoA: principal coordinates analysis
PLD: pelagic larval duration
POD: pseudo-observed dataset
Pool-seq: pooled DNA sequencing
RAD-seq: restriction site associated DNA sequencing
RDA: redundancy analysis
SNP: single nucleotide polymorphism
SSS: sea surface salinity
SSSmean: mean sea surface salinity
SSSrange: range in sea surface salinity
SST: sea surface temperature
SSTmean: mean sea surface temperature
SSTrange: range in sea surface temperature
Trange: range in air temperature
VIF: variance inflation factor
