# Supplementary material for: Multi-model seascape genomics identifies distinct environmental drivers of selection among sympatric marine species
Source: BMC Evol Biol. 2020 Sep 16;20:121. doi: 10.1186/s12862-020-01679-4 (PMC7493327; doi:10.1186/s12862-020-01679-4)
Supplement: Supplementary file 1 — Additional file 1. Sampling information, bioinformatic pipeline parameters, results of mitogenome mapping and de novo assembly comparisons, and single nucleotide polymorphism (SNP) results are shown per species. The study species distributions, and population clustering based on three coverage cut-off scenarios are also shown. [file 12862_2020_1679_MOESM1_ESM.docx]

**Additional File 1: Sampling information, bioinformatic pipeline parameters, results of mitogenome mapping and de novo assembly comparisons, and single nucleotide polymorphism (SNP) results are shown per species. Population structure based on three coverage scenarios are also shown per species. Species distributions are shown in comparison to the regional sea surface temperature gradient.**

**Table S1.** Sample sites with the corresponding abbreviation, date of collection, and number of collected individuals for *Cyclograpsus punctatus*.

| Sample Site | Abbreviation | Date collected | N |
| --- | --- | --- | --- |
| Jacobsbaai | JB | Aug 2015 | 38 |
| Yzerfontein | YZ | July 2018 | 40 |
| Sea Point | SP | July 2015 | 40 |
| Bettys Bay | BT | July 2018 | 40 |
| Gansbaai | GB | July 2018 | 40 |
| Cape Agulhas | CA | Aug 2015 | 40 |
| Jongensfontein | JF | July 2018 | 40 |
| Mossel Bay | MB | July 2018 | 40 |
| Knysna | KY | Aug 2015 | 40 |
| Cape St. Francis | CF | Aug 2015 | 37 |
| Haga Haga | HH | Oct 2015 | 40 |
| Coffee Bay | CB | July 2018 | 39 |
| Margate | MG | Nov 2018 | 40 |

**Table S2.** Sample sites with the corresponding abbreviation, date of collection and number of collected individuals for *Parechinus angulosus.*

| Sample Site | Abbreviation | Date collected | N |
| --- | --- | --- | --- |
| Port Nolloth | PN | July 2015 | 40 |
| Hondeklipbaai | HB | July 2015 | 40 |
| Doringbaai | DB | Aug 2015 | 40 |
| Lambertsbaai | LB | July 2015 | 40 |
| Jacobsbaai | JB | July 2015 | 40 |
| Sea Point | SP | July 2015 | 40 |
| Bettys Bay | BT | July 2018 | 40 |
| Cape Agulhas | CA | Aug 2015 | 39 |
| Mossel Bay | MB | July 2018 | 39 |
| Knysna | KY | Aug 2015 | 40 |
| Cape St. Francis | CF | Oct 2015 | 40 |
| Port Alfred | PA | July 2018 | 40 |
| Haga Haga | HH | Oct 2015 | 40 |
| Coffee Bay | CB | July 2018 | 40 |

**Table S3.** Sample sites with the corresponding abbreviation, date of collection and number of collected individuals for *Scutellastra granularis*.

| Sample Site | Abbreviation | Date collected | N |
| --- | --- | --- | --- |
| Port Nolloth | PN | July 2015 | 40 |
| Hondeklipbaai | HB | July 2015 | 40 |
| Brandsebaai | BB | July 2015 | 40 |
| Lambertsbaai | LB | July 2015 | 40 |
| Jacobsbaai | JB | July 2015 | 40 |
| Sea Point | SP | July 2015 | 40 |
| Bettys Bay | BT | July 2018 | 40 |
| Cape Agulhas | CA | Aug 2015 | 40 |
| Mossel Bay | MB | July 2018 | 39 |
| Knysna | KY | Aug 2015 | 40 |
| Cape St. Francis | CF | Oct 2015 | 40 |
| Port Alfred | PA | July 2018 | 40 |
| Haga Haga | HH | Oct 2015 | 40 |
| Hluleka | HL | July 2018 | 30 |

**Table S4.** The bioinformatic steps used to generate SNP datasets per species, with the associated program and parameters.

| Step | Program | Parameters |
| --- | --- | --- |
| 1. Filter raw reads | TrimGalore! | - q= 20 - length =50 - a/a2 = adapter sequences - stringency = 10 - e = 0.01 |
| 1. Map reads onto mitogenomes | BWA-MEM | - a - T=20 |
| 1. Convert SAM to BAM format | SAMtools | - default |
| 1. Filter mtDNA-mapped BAMs | SAMtools | - F 0X0004 - q = 10 |
| 1. Merge mtDNA-mapped BAMs | BAMtools | - default |
| 1. Convert merged BAMs to SAM files | SAMtools | - default |
| 1. Filter mtDNA-mapped reads from quality-trimmed reads | BBMap | - include = f - minlen = 50 |
| 1. Normalize reads for de novo assemblies | BBMap | - tossbadreads = t - target = 100 |
| 1. Identify k-mers for de novo assemblies | KmerGenie | - default |
| 1. AbySS assembly | Abyss | - c = 3 |
| 1. Megahit assembly | Megahit | - default |
| 1. Spades assembly | Spades | - careful |
| 1. GARM merge Spades assemblies | GARM | - default |
| 1. Compare assemblies | QUAST  BUCSO  NCBI BLAST + | - default - default using metazoan database - default using nucleotide database |
| 1. Map reads onto de novo assembly | BWA-MEM | - a - T=20 |
| 1. Convert SAM to BAM format | SAMtools | - default |
| 1. Filter mapped BAMs | SAMtools | - F 0X0004 - F 0X0008 - f  0X0002 - q = 20 |
| 1. Subsample BAMs to median # of quality-filtered mapped reads | SAMtools | - default |
| 1. Create a multiple-pileup file | SAMtools | - B - Q = 20 |
| 1. Create a sync file | PopPoolation2 | - min-qual = 20 |
| 1. Call SNPs | Poolfstat | - min.cov.per.pool = 20 - max.cov.per.pool = 400 - min.maf = 0.01 - min.rc = 4 |

**Table S5.** Reference mitogenomes, their size, and average number of raw reads per sample that mapped to the mitogenomes, shown for *C. punctatus*, *P. angulosus*, and *S. granularis*.

|  | *C. punctatus* | *P. angulosus* | *S. granularis* |
| --- | --- | --- | --- |
| Reference mitogenome / Accession # | *Cyclograpsus granulosus* / NC_025571.1 | *Loxechinus albus* / JX888466.1 | *Lottia digitalis* / DQ238599.1 |
| Reference mitogenome size (in base pairs; bp) | 16,300 bp | 15,260 bp | 26,835 bp |
| Average number of reads per sample mapped onto mitogenome | 12,363 | 20,342 | 234 |

**Table S6.** Quast outputs from three different de novo assembly methods (AbySS, MEGAHIT, and SPAdes) shown for *C. punctatus, P. angulosus*, and *S. granularis.*

| Quast output | AbySS | MEGAHIT | SPAdes |
| --- | --- | --- | --- |
| *C. puntatus* | | | |
| N50 | 713 | 576 | 1,609 |
| L50 | 475 | 537 | 755 |
| # contigs > 1000 bp | 164 | 21 | 1,592 |
| Largest contig | 4,174 | 1,691 | 8,615 |
| Total length | 955,536 | 747,360 | 3,813,367 |
| BLASTN % nodes matched | 0.01 | 0.06 | 0.20 |
| *P. angulosus* | | | |
| N50 | 659 | 614 | 1,434 |
| L50 | 8,555 | 7,399 | 1,309 |
| # contigs > 1000 bp | 1,567 | 552 | 2,432 |
| Largest contig | 5,354 | 2,116 | 10,665 |
| Total length | 14,836,022 | 11,240,686 | 5,791,195 |
| BLASTN % nodes matched | 0.04 | 0.10 | 0.19 |
| *S. granularis* | | | |
| N50 | 553 | 586 | 886 |
| L50 | 67 | 7,933 | 6,307 |
| # contigs > 1000 bp | 0 | 156 | 4,505 |
| Largest contig | 806 | 1,965 | 7,721 |
| Total length | 83,626 | 10,866,182 | 16,058,421 |
| BLASTN % nodes matched | 0.01 | 0.03 | 0.07 |

**Table S7.** The number (#) of total raw and mapped reads, number of reads passing filtering, number of total SNPs and SNPs passing filtering, for *C. punctatus*, *P. angulosus* and *S. granularis*.

| Species | # Raw reads | # Mapped reads | # Reads after filtering | # Total SNPS | # SNPs after filtering |
| --- | --- | --- | --- | --- | --- |
| *C. punctatus* | 29,246,761 | 23,431,343 | 20,641,431 | 17,309 | 1,190 |
| *P. angulosus* | 47,663,829 | 33,686,426 | 20,570,929 | 3,946 | 822 |
| *S. granularis* | 39,463,412 | 29,734,860 | 27,443,789 | 10,416 | 1,658 |

**Table S8.** Site-specific number (#) of raw reads, quality-mapped reads, and total filtered SNPs, and mean depth coverage per pool are shown for *C. punctatus*.

| Site | # Raw reads | # Mapped reads | # of SNPs | Mean depth coverage |
| --- | --- | --- | --- | --- |
| JB | 1,255,227 | 1,094,399 | 682 | 60.7 |
| YZ | 655,848 | 566,740 | 552 | 39.7 |
| SP | 2,691,816 | 2,395,053 | 908 | 138.4 |
| BT | 2,656,600 | 2,132,438 | 995 | 158.1 |
| GB | 2,443,284 | 1,883,225 | 976 | 149.7 |
| CA | 2,690,586 | 2,109,919 | 955 | 152.5 |
| JF | 2,431,546 | 1,964,114 | 928 | 144.9 |
| MB | 2,813,579 | 2,169,307 | 946 | 141.2 |
| KY | 1,132,946 | 971,259 | 712 | 65.8 |
| CF | 1,268,052 | 1,003,981 | 760 | 76.1 |
| HH | 4,703,307 | 3,674,480 | 962 | 148.1 |
| CB | 3,718,276 | 2,963,114 | 982 | 159.1 |
| MG | 785,694 | 503,314 | 448 | 37.2 |

**Table S9.** Site-specific number (#) of raw reads, quality-mapped reads, and total filtered SNPs, and mean depth coverage per pool are shown for *P. angulosus.*

| Site | # Raw reads | # Mapped reads | # of SNPs | Mean depth coverage |
| --- | --- | --- | --- | --- |
| PN | 2,278,589 | 1,614,913 | 520 | 110.9 |
| HB | 1,751,423 | 1,261,453 | 484 | 76.9 |
| DB | 2,316,543 | 1,779,152 | 590 | 116.5 |
| LB | 2,226,368 | 1,604,554 | 631 | 125.7 |
| JB | 3,237,292 | 2,461,858 | 612 | 129.2 |
| SP | 1,809,007 | 1,033,637 | 441 | 57.3 |
| BT | 862,961 | 655,017 | 375 | 52.0 |
| CA | 1,737,662 | 1,296,096 | 491 | 90.3 |
| MB | 2,588,031 | 1,880,341 | 551 | 116.3 |
| KY | 17,984,447 | 13,096,132 | 577 | 115.8 |
| CF | 2,369,908 | 1,478,938 | 364 | 55.9 |
| PA | 2,526,174 | 1,860,987 | 572 | 107.0 |
| HH | 3,242,187 | 1,706,892 | 513 | 99.2 |
| CB | 2,733,237 | 1,956,456 | 821 | 110.9 |

**Table S10.** Site-specific number (#) of raw reads, quality-mapped reads, and total filtered SNPs, and mean depth coverage per pool are shown for *S. granularis.*

| Site | # Raw reads | # Mapped reads | # of SNPs | Mean depth coverage |
| --- | --- | --- | --- | --- |
| PN | 2,458,390 | 2,098,877 | 1,073 | 85.3 |
| HB | 3,068,493 | 2,320,497 | 1,157 | 105.3 |
| BB | 3,347,812 | 2,449,271 | 1,217 | 111.7 |
| LB | 2,574,014 | 1,894,418 | 1,178 | 99.9 |
| JB | 3,227,994 | 2,345,470 | 1,196 | 113.4 |
| SP | 3,051,925 | 2,420,161 | 1,151 | 108.4 |
| BT | 4,745,447 | 3,685,996 | 1,223 | 114.3 |
| CA | 886,627 | 629,450 | 756 | 38.3 |
| MB | 1,146,977 | 813,173 | 793 | 51.6 |
| KY | 3,142,000 | 2,327,798 | 1,187 | 106.0 |
| CF | 2,708,281 | 2,065,118 | 1,189 | 109.2 |
| PA | 1,253,857 | 904,309 | 794 | 53.5 |
| HH | 1,179,705 | 908,600 | 798 | 45.9 |
| HL | 6,671,890 | 4,871,722 | 1,121 | 120.6 |


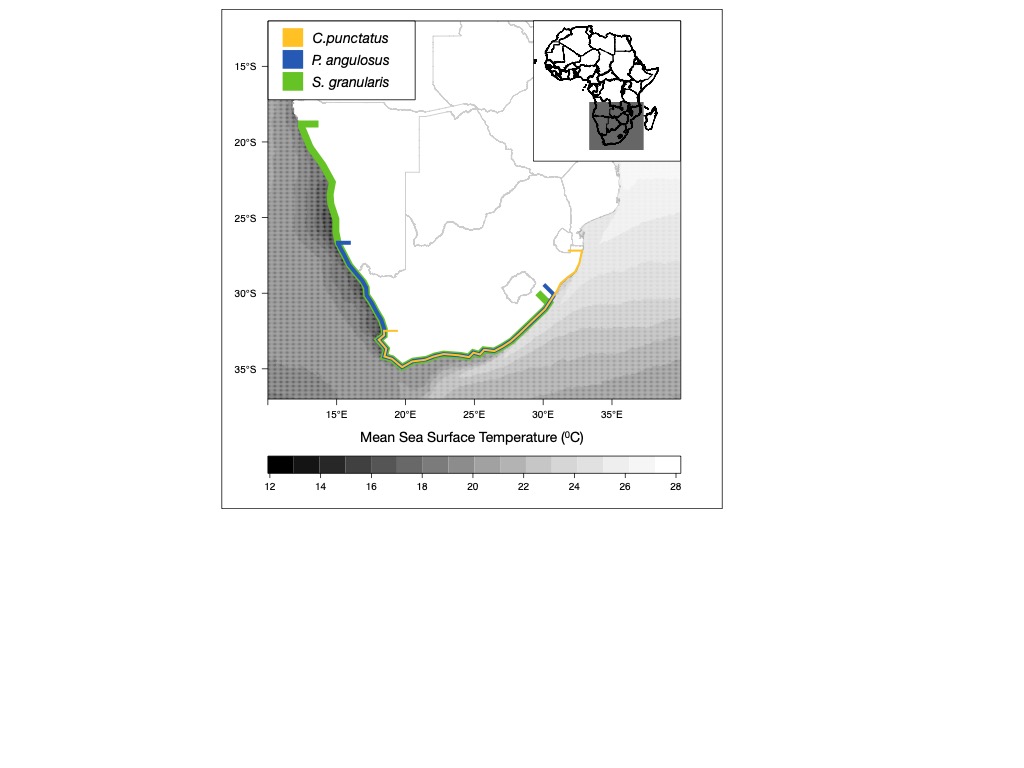


**Figure S1**. The species distributions with *C. punctatus* in yellow, *P. angulosus* in blue and *S. granularis* is green, shown over the average sea surface temperatures from the MARSPEC database [1].


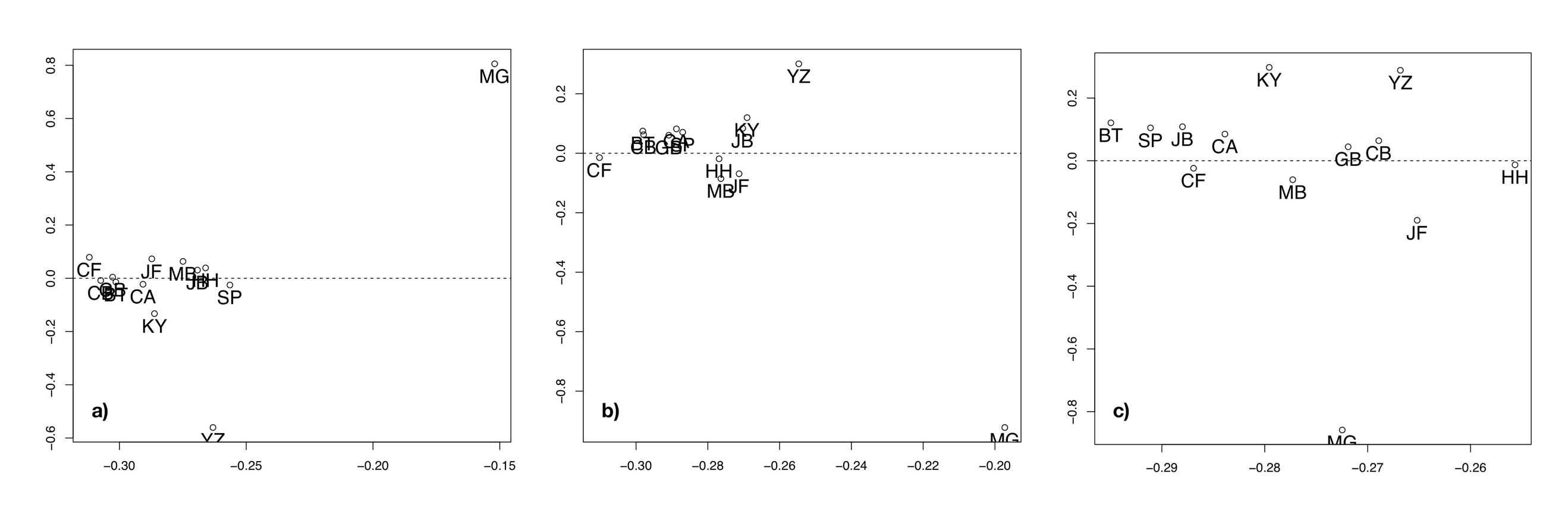


**Figure S2.** Clustering of *C. punctatus* populations derived from a singular value decomposition of the scaled covariance matrices of population allele frequencies (Ω) from the core Bayesian hierarchical model implemented in BayPass [2], shown for three coverage parameter scenarios: minimum coverage = 20, maximum coverage = 200 (a); minimum coverage = 20, maximum coverage = 400 (b); minimum coverage = 40, maximum coverage = 400 (c). Refer to Table S1 for sample site abbreviations.


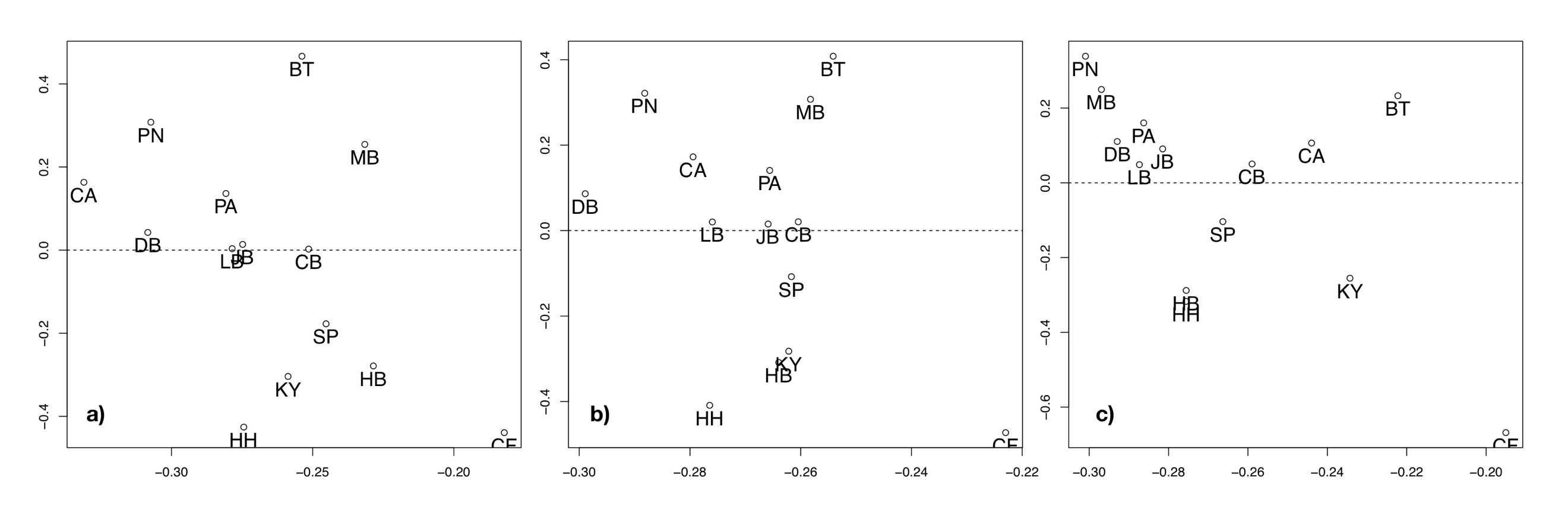


**Figure S3**. Clustering of *P. angulosus* populations derived from a singular value decomposition of the scaled covariance matrices of population allele frequencies (Ω) from the core Bayesian hierarchical model implemented in BayPass [2], shown for three coverage parameter scenarios: minimum coverage = 20, maximum coverage = 200 (a); minimum coverage = 20, maximum coverage = 400 (b); minimum coverage = 40, maximum coverage = 400 (c). Refer to Table S2 for sample site abbreviations.


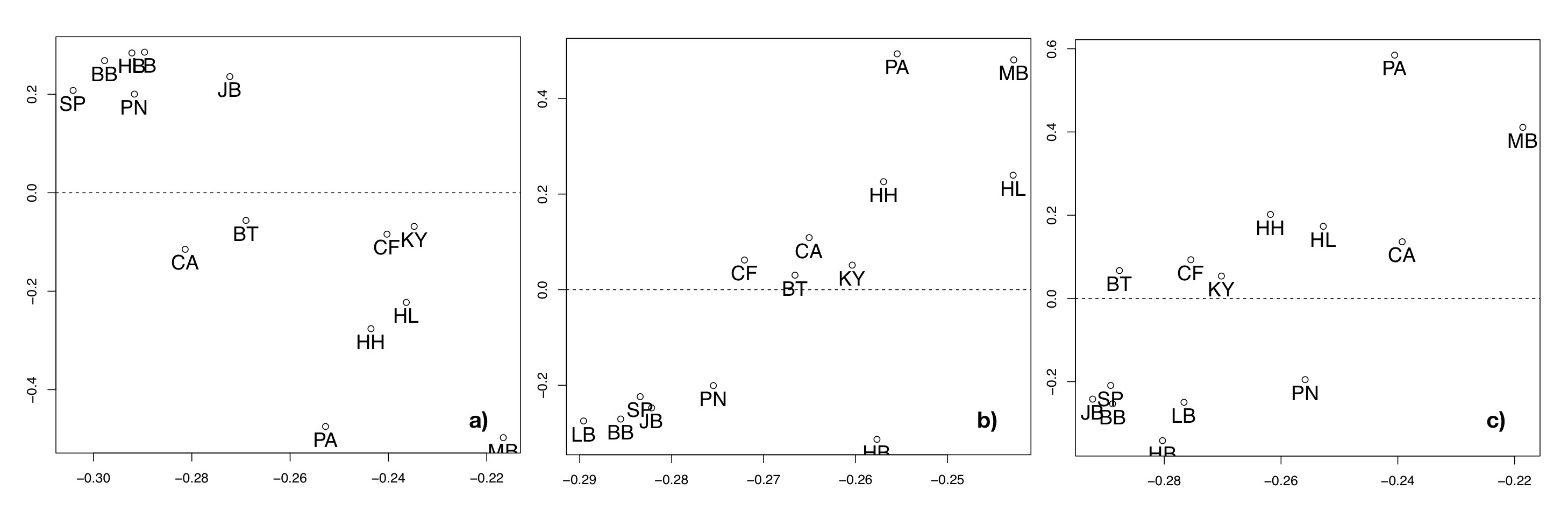


**Figure S4.** Clustering of *S. granularis* populations derived from a singular value decomposition of the scaled covariance matrices of population allele frequencies (Ω) from the core Bayesian hierarchical model implemented in BayPass [2], shown for three coverage parameter scenarios: minimum coverage = 20, maximum coverage = 200 (a); minimum coverage = 20, maximum coverage = 400 (b); minimum coverage = 40, maximum coverage = 400 (c). Refer to Table S3 for sample site abbreviations.

Literature Cited

1. Sbrocco EJ, Barber PH. MARSPEC: ocean climate layers for marine spatial ecology. Ecology. 2013;94(4):979–979.

2. Gautier M. BAYPASS version 2.1 User Manual. 2015.
